# TMPyP4 promotes cancer cell migration at low doses, but induces cell death at high doses

**DOI:** 10.1038/srep26592

**Published:** 2016-05-25

**Authors:** Xiao-Hui Zheng, Xin Nie, Hai-Ying Liu, Yi-Ming Fang, Yong Zhao, Li-Xin Xia

**Affiliations:** 1 Medical School, Shenzhen University, Shenzhen 518060, P. R. China; 2 Key Laboratory of Gene Engineering of the Ministry of Education, School of Life Sciences, Sun Yat-sen University, Guangzhou 510006, P. R. China; 3 Collaborative Innovation Center of High Performance Computing, National University of Defense Technology, Changsha 410073, P. R. China

## Abstract

TMPyP4 is widely considered as a potential photosensitizer in photodynamic therapy and a G-quadruplex stabilizer for telomerase-based cancer therapeutics. However, its biological effects including a possible adverse-effect are poorly understood. In this study, whole genome RNA-seq analysis was used to explore the alteration in gene expression induced by TMPyP4. Unexpectedly, we find that 27.67% of changed genes were functionally related to cell adhesion. Experimental evidences from cell adhesion assay, scratch-wound and transwell assay indicate that TMPyP4 at conventional doses (≤0.5 μM) increases cell-matrix adhesion and promotes the migration of tumor cells. In contrast, a high dose of TMPyP4 (≥2 μM) inhibits cell proliferation and induces cell death. The unintended “side-effect” of TMPyP4 on promoting cell migration suggests that a relative high dose of TMPyP4 is preferred for therapeutic purpose. These findings contribute to better understanding of biological effects induced by TMPyP4 and provide a new insight into the complexity and implication for TMPyP4 based cancer therapy.

Photodynamic therapy (PDT) induces cancer cell death (necrosis or apoptosis) mainly by reactive oxygen species (ROS), which are produced by irradiated photosensitizers^1^. Compared to the conventional anticancer therapy, PDT is less invasive with better tolerance and outcome. In addition, PDT has obvious advantages over other cancer therapeutics such as surgery, radiation and chemotherapy: a minimal functional disturbance, being repetitively applicable on the same site and a low recurrence^2^. PDT has been rapidly developed over past decades with a great potential to treat multiple types of cancers including esophageal cancer and non-small cell lung cancer^3^,^4^. The photosensitizer is crucial for PDT treatment^5^. However, it has been challenging to obtain an optimal photosensitizer with a high yield of singlet oxygen (^1^O_2_) and high precision targeting cancer cells^5^. TMPyP4 (Fig. 1A), a porphyrins derivative, has been considered as a promising photosensitizer due to its high water solubility, high permeability through cell membrane and preferential accumulation in tumor cells^6^,^7^,^8^.

Besides potentially serving as a photosensitizer in PDT, TMPyP4 has been recently developed as a chemotherapeutics drug to inhibit telomerase activity in cancer cells^9^,^10^,^11^. About 85% of cancer cells overcome the proliferative limit by activating telomerase, a ribonucleoprotein with reverse transcriptase activity that adds telomeric DNA repeats to the 3′-overhang of telomeres, thus maintaining telomere length and chromosome integrality^12^. Accumulated evidences show that single-stranded 3′-overhang of telomeres can stack via Hoogsteen hydrogen bonding into a structure referred as G-quadruplex^13^. TMPyP4 is able to associate and stabilize G-quadruplex, thereby blocking telomerase action. TMPyP4 treatment leads to progressive telomere shortening that eventually results in cancer cell death by apoptosis or senescence^14^.

Because DNA sequence with a potential to form G-quadruplex is widely present on genome, it has been reported that TMPyP4 treatment may lead to multiple consequences including the alteration of expression of particular genes^15^,^16^,^17^,^18^,^19^,^20^ and/or the interference with DNA replication^21^,^22^. Therefore, it is important to comprehensively understand biological effects induced by TMPyP4 before it can be used for anti-cancer therapeutics. Moreover, a possible adverse effect is worth investigating.

In this report, human A549 cancer cells were treated with TMPyP4 or its derivative TPyP4-Pt (Fig. 1B), and gene expression profile for treated and untreated cells was obtained by RNA-seq. Unexpectedly, we found that among the genes changed by TMPyP4 or TPyP4-Pt, ~27% are involved in cell adhesion and migration, implying that TMPyP4 treatment might affect cancer metastasis. The experiments including cell adhesion assay, scratch-wound healing assay and transwell assay demonstrate that TMPyP4 at commonly used dose (≤0.5 μM, close to its light *IC*_50_ values) promotes cancer cell migration. In strikingly contrast, the high-dose of TMPyP4 (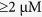) inhibits cell proliferation and induces cell death. These findings provide new insights into the complexity of TMPyP4 as a possible anticancer drug.

## Results

### TMPyP4 changes the expression of adhesion-related genes in human lung cancer cells A549

**Figure 1:**
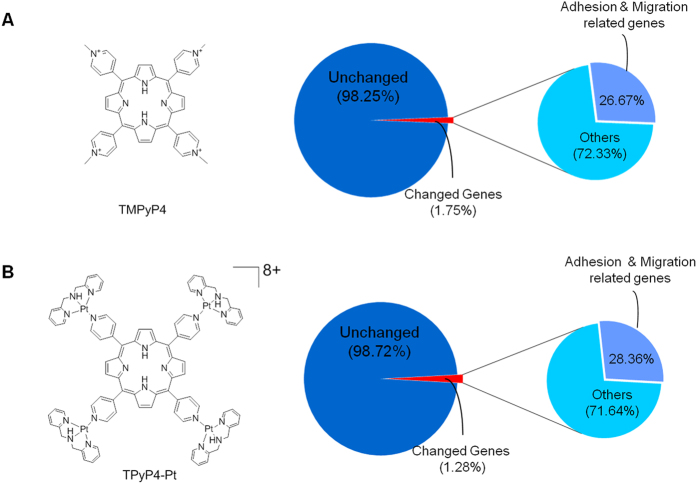
TMPyP4 or TPyP4-Pt treatment results in the change of gene expression profile in A549 cells. (**A**) The molecule of TMPyP4 used in this study. Counter ions are p-toluenesulfonate. Statistics of RNA-seq data comparing gene expression in TMPyP4 treated and untreated A549 cells. “GO” analysis showed the functional group of genes changed in expression. (**B**) As in (**A**) except that TPyP4-Pt was used to treat cells. Counter ions are NO_3_^−^ anions.

The effect of TMPyP4 on global gene expression in cancer cells was evaluated using RNA-seq, a whole transcriptome sequencing (mRNA, Hiseq2000-PE125). Human A549 lung cancer cells were cultured in the presence or absence of 0.5 μM TMPyP4 for 2 days; their mRNA was isolated and subjected to RNA-seq. The top 100 changed mRNA transcripts and their abundance are listed in Table S1 and full sequence data from these experiments were uploaded to GEO database under accession number of GSE72983. Changed genes were functionally grouped by GO-biology analysis. Our results showed that the expression of 1.73% genes was changed upon TMPyP4 treatment. 27.67% genes of them were functionally related to cell adhesion and migration (Fig. 1A, Table S4). To further verify these data, we treated A549 cells with TPyP4-Pt (Fig. 1B), a derivative of TMPyP4 with similar characteristics to TMPyP4^15^. Consistent with the results from TMPyP4, TPyP4-Pt treated cells displayed the increasement of cell adhesion and migration related genes (28.36%) (Fig. 1B, Table S2 and Table S5).

### TMPyP4 increases cell-matrix adhesion

**Figure 2:**
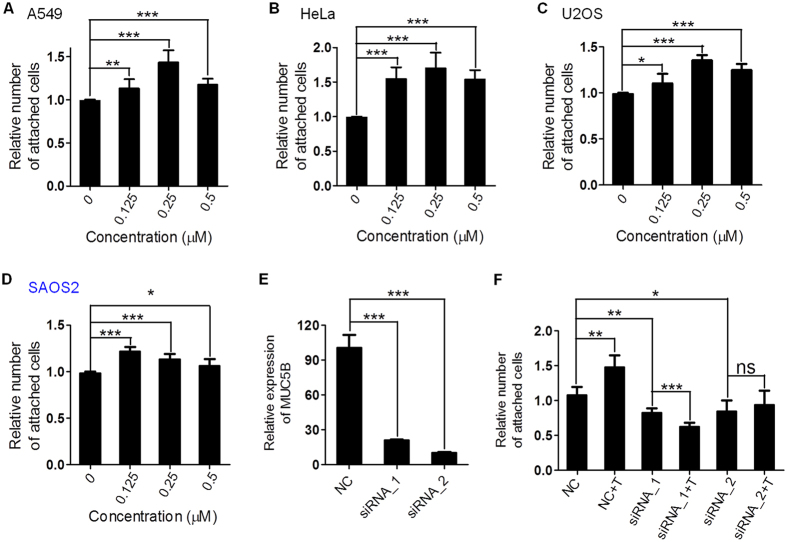
The effect of TMPyP4 on cells adhesion to extracellular matrix. (**A–D**) TMPyP4 increase cell adhesion to extracellular matrix. Indicated cell lines were used. (**E**) qRT-PCR showed the abundance of MUC5B mRNA in A549 cancer cell line (NC) and A549 cells transfected with siRNA targeting MUC5B (siRNA_1 and siRNA_2). (**F**) MUC5B knock-down suppress the adhesion of A549 cancer cells to extracellular matrix. MUC5B deficient cells showed no increase of cell adhesion upon TMPyP4 treatment. Values are average ± SD of three independent experiments.

The commonly used concentration for TMPyP4 study is near its light *IC*_50_ values, i.e. 0.25 μM *in vitro*^23^. RNA-seq results implied that cell adhesion to extracellular matrix may be changed upon TMPyP4 or TPyP4-Pt treatment. To test this, cell adhesion assay that determines the adhesion between cell and attached matrix was performed in multiple cancer cell lines including human A549, HeLa, osteosarcoma U2OS and SAOS2. As shown in Fig. 2, all four cell lines displayed significant increase in cell adhesion to matrix after treatment with TMPyP4 (0.125 μM, 0.25 μM and 0.5 μM). Similar phenomena were observed when cells were treated with TPyP4-Pt (Figure S1). These results suggested that TMPyP4 and its derivate increases cell-matrix adhesion by changing the expression of adhesion-related genes. To validate this, MUC5B, a top hit of RNA-seq that has been previously reported to promote cell adhesion^24^, was knocked down by siRNA in A549 cells (Fig. 2E). Indeed, the knockdown of MUC5B resulted in the decrease of cell attachment (Fig. 2F). However, TMPyP4 failed in promoting the attachment of MUC5B deficient cells (Fig. 2F), demonstrating that the change of gene expression such as MUC5B is responsible for increased cell adhesion upon TMPyP4 treatment.

### TMPyP4 promotes cancer cell migration

**Figure 3:**
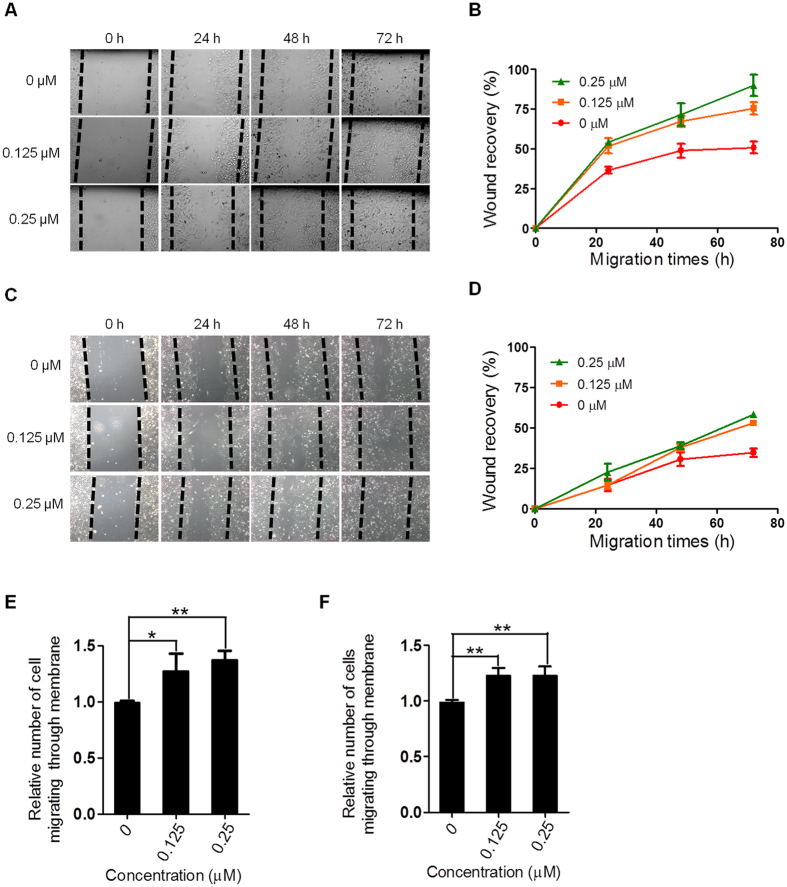
The effect of TMPyP4 on cell migration rate. (**A**) TMPyP4 promotes A549 cells migration in scratch-wound healing assay. (**B**) Quantification of (**A**). (**C**) as in (**A**) except U2OS cells were used. (**D**) Quantification of (**C**). (**E**) TMPyP4 promoted A549 cells migration in a transwell assay. (**F**) as in (**E**) except U2OS cells were used. Values are average ± SD of three independent experiments.

Cell adhesion is often associated with cell migration^25^. To this end, we performed scratch-wound healing assay to determine the migration rate of A549 and U2OS cells in the presence or absence of TMPyP4. Our results showed that TMPyP4 significantly increased cancer cell migration (Fig. 3). After 72h treatment, 51% scratch was covered by migrated A549 cells, while 90% scratch was covered when cells were treated with 0.25 μM TMPyP4 (Fig. 3A,B). Similarly, 0.25 μM TMPyP4 treated U2OS cells showed significantly increased migration rate (Fig. 3C,D). TPyP4-Pt treated A549 cells also displayed increased cell migration compared to untreated cells (Figure S2). These results demonstrated that TMPyP4 and its derivate enhance the migration of cancer cells.

To further confirm the strengthening migration ability of cancer cells induced by TMPyP4, transwell migration assay was performed, in which cells are initially placed on the upper layer of permeable cell membrane and the cells that have migrated through the membrane are stained and counted after certain period of times. The quantitative data demonstrated that A549 and U2OS cells treated with TMPyP4 exhibited increased transferability compared to untreated cells (Fig. 3E,F).

### TMPyP4 at high dose induce cancer cell death

**Figure 4:**
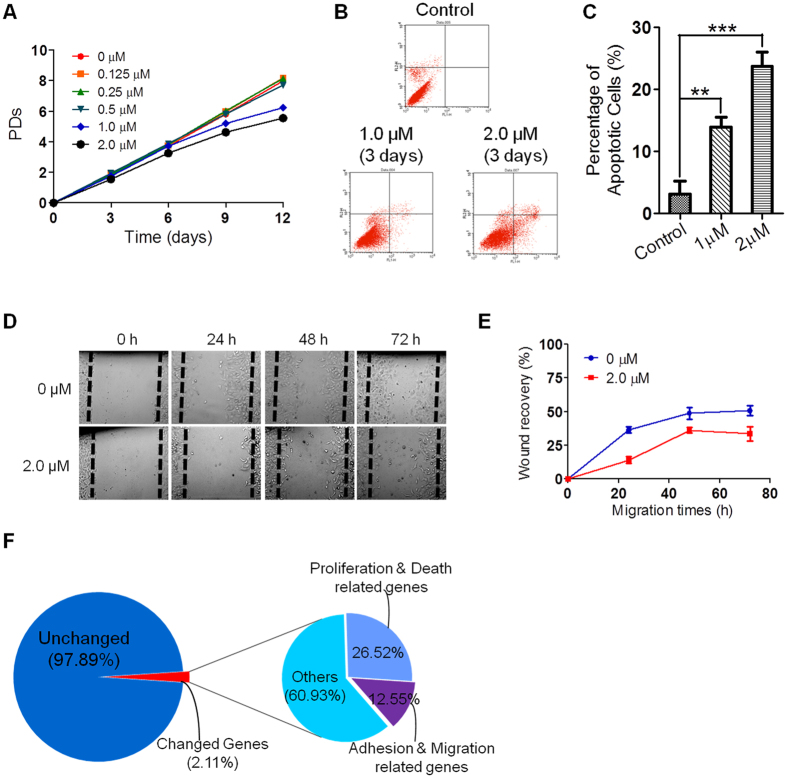
The effect of high dose TMPyP4 on proliferation, cell apoptosis, migration and gene expression. (**A**) Proliferation curve of A549 cells in the presence of low and high dose of TMPyP4. (**B**) 1.0 or 2.0 μM TMPyP4 treatment induces apoptosis of A549 cells. Apoptotic cells were assayed by Annexin V/PI staining and FACS analysis. (**C**) Quantification of (**B**). (**D**) Scratch-wound healing assay showing decreased migration rate of A549 cells when treated with high-dose TMPyP4 (2.0 μM). (**E**) Quantification of (**D**). (**F**) Statistics of RNA-seq data comparing gene expression in 2.0 μM TMPyP4 treated and untreated A549 cells. Values are average ± SD of three independent experiments.

While TMPyP4 at commonly used concentration (0.25 or 0.5 μM) may have undesired “side-effect” on promoting cancer cell migration, we then explored a possible strategy to eliminate this effect. It has come to our attention that A549 cells treated with higher concentration of TMPyP4 (2 μM) exhibited remarkably slower proliferation compared with control cells or cells treated with lower dose of TMPyP4 (Fig. 4A). This phenomenon was also observed in HeLa, U2OS and SAOS2 cells (Figure S3). FACS analysis indicated that 2.0 μM TMPyP4 induced apoptosis of ~23% A549 cells after only 3 days (Fig. 4B,C). Likewise, 33% U2OS cells underwent apoptosis after 3 days treatment with 2.0 μM of TMPyP4 (Figure S4). Moreover, scratch-wound healing assay demonstrated that the treatment with 2.0 μM TMPyP4 slowed down cell migration rate (Fig. 4D,E and Figure S5).

As expected, TMPyP4 inhibited telomerase activity of A549 (Figure S6), however, it is unlikely that rapid cell apoptosis (in 3 days) induced by TMPyP4 is due to the inhibition of telomerase. We thus performed RNA-seq analysis to determine the change of gene expression in response to high dose of TMPyP4 (2 μM). The results showed that expression of 2.11% genes was changed upon TMPyP4 (2 μM) treatment (Fig. 4F and Table S3), of which 26.52% genes were functionally related to cell proliferation and apoptosis, and 12.55% genes were related to cell adhesion and migration (Fig. 4F, Table S3 and Table S6). Strikingly, a substantial number of cell adhesion and migration related genes are found in both 0.5 and 2 μM treated cells, indicating the same mechanism underlying gene regulation by TMPyP4.

## Discussion

TMPyP4 treatment changes the expression of a significant number of genes (1.73% of whole genome). TMPyP4 and its derivate may affect gene transcription in multiple manners. First, TMPyP4 is able to incorporate into duplex DNA^26^, thus may facilitate or block gene transcription; second, given the fact that G-rich sequences that can fold into G-quadruplex are widely present on genome^27^ and highly enriched in promoter region of genes^28^, it is conceivable that G-quadruplex stabilized by TMPyP4 interferes the transcription of those genes; third, TMPyP4 may increase the level of ROS in cells (even cells are cultured in dark, the light can’t be absolutely excluded), thereby resulting in a global cell response including the change in expression of particular genes. Intriguing finding in this study is that among all genes affected by TMPyP4 (~1.7% of whole genome), ~27% of them are involved in cell adhesion and migration (Fig. 1A). While molecular mechanisms underlying the regulation of these genes are remained to be elucidated, the consequence is that TMPyP4 treated cells exhibit increased ability to adhere to extracellular matrix (Fig. 2). Accordingly, increased migration rate (Fig. 3A–D) and transferability (Fig. 3E,F) was observed for TMPyP4 treated cells. It has been widely accepted that increased cell migration and transferability are closely associated with cancer invasion and metastasis, a major cause of patient death for variety of cancers^29^. Thus, our study implies that TMPyP4 may increase the risk of cancer metastasis. This raises a serious question regarding how to prevent this “side-effect” of TMPyP4 in cancer therapeutics.

TMPyP4 is first developed as a photosensitizer in PDT^6^,^7^,^8^. The treatment process of PDT involves the injection of photosensitizer to patient’s blood^7^, followed by irradiation of diseased tissue (tumors) with a high intensity light^7^. However, a number of tumors may escape from irradiation during treatment of metastatic cancers due to the fact that PDT cannot irradiate all the tumors at a time^30^,^31^. In addition, the light used in PDT cannot penetrate more than 1cm tumor tissue^30^,^31^. In these scenarios, escaped cancer cells may become more metastatic aggressive. Our results thus provide a strong rational to pay a great attention to the “side-effect” of TMPyP4 in PDT.

TMPyP4 has also been proposed to serve as a potential G-quadruplex stabilizer to inhibit telomerase-mediated telomere lengthening^9^,^10^,^32^. Because telomerase is exclusively present in cancer and stem cells, the inhibition of telomerase action has been considered as a promising approach for specifically targeting cancer cells. As telomeres shorten at rate of ~60 bp/PD in absence of telomerase, it takes a long time before cancer cells undergo crisis due to extremely short telomeres^33^. In this context, TMPyP4 may not be a good candidate for telomerase-based therapeutics because it increases the risk of metastasis.

The treatment with a high dose of TMPyP4 (≥2 μM) results in cell apoptosis, which may be responsible for slower cell proliferation and decreased migration rate (Fig. 4). High doses may be toxic to normal cells in body; it nevertheless provides a possible option for application of TMPyP4 in oncotheropeutics. Alternatively, TMPyP4 needs to be used in combination with other therapeutic drug to minimize its side-effect. Altogether, our study reveals dose-dependent effect of TMPyP4 in cancer treatment and provides a strong rational to re-consider the strategy for application of TMPyP4 in cancer therapeutics.

## Methods

### Cell Culture

A549, HeLa, U2OS and SAOS2 were obtained from Cell Resource Center of Peking Union Medical College and cultured at 37 °C under 5% CO_2_ in DMEM (gibco) supplemented with 10% fetal calf serum (gibco) and 100 U/mL penicillin and streptomycin (HyClone). TMPyP4 was purchased from Sigma.

### RNA-seq whole transcriptome sequencing

8.0 × 10^5^ A549 cells were seeded to 10 cm^2^ dish, after incubating for 6 h, the TMPyP4 or TPyP4-Pt was added to medium (a final concentration of 0.5 μM). After incubating for 2 days, cells were harvested and total RNA were isolated by Trizol (Takara) extraction. 3 μg RNA per sample was used as input material for the RNA sample preparations. Sequencing libraries were generated using a NEBNext Ultra RNA Library Prep Kit for Illumina (NEB, USA) according to the manufacturer’s recommendations and sequencing was performed on an Illumina Hiseq 2000 platform. Raw data were extracted by Basecalls of CASAVA (version 1.8). Sequenced reads were mapped to whole genome (hg19) using Tophat2.0.2. Unique reads were used for further analysis. The abundance of reads for genes was determined by htseq-count script (Version 0.6.1) with parameters -s no. The expression level of genes was normalized by Fragment Per Kilobase of exon per Megabase of library size (FPKM)(50). The correlation between different samples was measured by Spearman’s rank correlation test and visualized with R package.

### Long-term cell proliferation

Long-term cell proliferation experiments were carried out using A549, HeLa, U2OS and SAOS2 cancer cell lines. 5.0 × 10^5^ cells were seeded to 10 cm^2^ dish and incubated for 6 h, TMPyP4 or TPyP4-Pt was added to medium to a final concentration of 0.125, 0.25, 0.5, 1.0 or 2.0 μM, respectively. The medium was changed every three days until confluence was reached.

### Annexin V/PI apoptosis assay

Cells were seeded in 10 cm^2^ dish at a density of 5.0 × 10^5 ^cells per dish and incubated at 37 °C for ~6 h until cells attached to the dish. TMPyP4 with final concentration of 1.0 or 2.0 μM were then added into medium. After 3 days, cells were harvested for anexin V/PI apoptosis assay. The assay was performed following the protocol provided by the Annexin V/PI apoptosis Kit (Sigma).

### Cell adhesion assay

The 96-well plate was coated with 2.5 μg/ml human fibronectin in PBS (Millipore, CA) for 2 h at room temperature. Cells were seeded into the 96-well plate at a density of 4 × 10^4 ^cells/well and cultured for 1h at 37 °C in a CO_2_ incubator. Cells were then rinsed three times with 10% formalin and stained with crystal violet for 5 min at room temperature. After three times washing with ddH_2_O, stained cells were dissolved in 100 μL 33% acetic acid. The absorbance at 560 nm was detected by Synergy H1 Multi-Mode Reader (BioTek). Relative number of cells attaching to extracellular matrix was calculated using the following equation: mean OD of treated cells/mean OD of control cells. Cells treated with vehicle (0.1% DMSO) were used as a control.

### Transwell assay

Transwell assay was performed using Transwell Kit (Corning Costar, NY) following manufacturer’s instructions. Briefly, cells were pretreated with different concentrations (0, 0.125 or 0.25 μM) of TMPyP4 for 3 days, trypsinized and seeded into Transwell Permeable Support (insert) pre-equilibrated with serum-free DMEM medium. For each group, 1 × 10^5^cells/insert were seeded and incubated in 100 μl serum-free DMEM medium. The insert was placed on 24-well plate containing 600 μl of DMEM medium with 10% FBS. After 24 h of culturing, cells on the upper surface of insert were removed with cotton-tipped swabs. And the cells on backside surface of insert were fixed with 10% formalin, stained with crystal violet for 5 min at room temperature, and washed three times with ddH_2_O. Stained cells were dissolved in 500 μL 33% acetic acid and their absorbance was detected at 560 nm by Synergy H1 Multi-Mode Reader (BioTek).

### The scratch-wound assay

The cells were seeded in a 6-well plate at a density of 2 × 10^5 ^cells/well and cultured in medium containing TMPyP4 or TPyP4-Pt for 3 days. A denuded area was created across the diameter of dish by a yellow tip. The cells were washed with PBS and incubated in a serum free medium. Phase-contrast images were taken at a time point of 0, 24, 48 and 72 h of incubation. Images were analyzed with Axiovision Rel.4.8 software. The percentage of areas covered by migrated cells (wound recovery) was calculated. Three independent experiments were carried out for quantification.

### Knockdown of MUC5B by siRNA

MUC5B or PCDH17 was knocked down in A549 cells by siRNA. siRNA sequence are: siRNA_1-GCAGCTACGTTCTGTCCAA; siRNA_2 GCGTGTTCCTCAACTCCAT; siRNA were transfected into cells using Lipofectamine RNAiMAX (Life Technologies) following manufacturer’s instruments. SiRNA with scramble sequence was used as a negative control (NC). Cells were assayed 60 h after transfection.

### Quantitative real-time PCR (qRT-PCR)

Total RNA of A549 cells treated with indicated siRNA was extracted by RNAiso Plus Reagent (Takara). cDNA was produced using Superscript III Reverse Transcriptase (Invitrogen) and random primers. qRT-PCR were performed using Fast SYBR Green PCR mastermix (Invitrogen) and specific primers (MUC5B: 5′-GCCTACGAGGACTTCAACGTC-3′, 5′-CCTTGATGACAACACGGGTGA-3′; β-actin: 5′-CATGTACGTTGCTATCCAGGC-3′, 5′-CTCCTTAATGTCACGCACGAT-3′). The relative abundance of MUC5B mRNA were calculated using the formula of 2^−ΔΔCT^ and normalized to an endogenous housekeeping gene (β-actin). Values are means 6 standard deviation from four to six independent samples.

### TRAP assay

A telomerase extract (1.0 μl, 200 cells) was prepared from A549 cells with NP-40 lysis buffer. Each reaction was performed in a final volume of 20 μl in a reaction mixture containing of 2.0 μL of 10× TRAP buffer, 1.6 μL of dNTP mix, 0.4 μL of TS primer (100 ng/mL), 0.8 μL Primer mix (100 ng/mL), 2.0 μL TSNT internal control primer (4.0 × 10^−11^M), 0.4 μL of RNase inhibitor (2 U/mL), 0.16 μL of Taq polymerase (5 U/mL), 5.0 μL of different concentrations of TMPyP4 and 6.64 μL of DEPC-treated water. The experiment was performed as previously reported^34^,^35^. Relative telomerase activity was calculated as a ratio of signal in TRAP ladder to signal of an internal control.

### Statistical analysis

The student’s 2-tailed unpaired t-test was used to determine statistical significance and the resulting P-values are indicated in figures (*P < 0.05; **P < 0.01; ***P < 0.001).

## Additional Information

**How to cite this article**: Zheng, X.-H. *et al*. TMPyP4 promotes cancer cell migration at low doses, but induces cell death at high doses. *Sci. Rep.* **6**, 26592; doi: 10.1038/srep26592 (2016).

## Supplementary Material

Supplementary Information
