# Supplementary material for: TMPyP4 promotes cancer cell migration at low doses, but induces cell death at high doses
Source: Sci Rep. 2016 May 25;6:26592. doi: 10.1038/srep26592 (PMC4879555; doi:10.1038/srep26592)
Supplement: Supplementary Information [file srep26592-s1.pdf]

# Supplementary Information

## TMPyP4 promotes cancer cell migration at low doses, but induces cell death at high doses

Xiao-Hui Zheng[a,b,c], Xin Nie[b,c], Hai-Ying Liu[b,c], Yi-Ming Fang[b,c], Yong Zhao[b,c]* and Li-Xin Xia[a]*

[a] Medical School, Shenzhen University, Shenzhen 518060, P. R.China;
[b] Key Laboratory of Gene Engineering of the Ministry of Education, School of Life Sciences, Sun Yat-sen University, Guangzhou 510006 P. R.China;
[c] Collaborative Innovation Center of High Performance Computing, National University of Defense Technology, Changsha 410073, P. R.China;
* To whom correspondence should be addressed: YZ, E-mail: zhaoy82@mail.sysu.edu.cn, Tel: 86-203-994-3401, Fax: 86-203-994-3778 or LX, Email: xialixin@126.com, Tel: 86-131-1362-1826, Fax: 86-755-8667-1913.

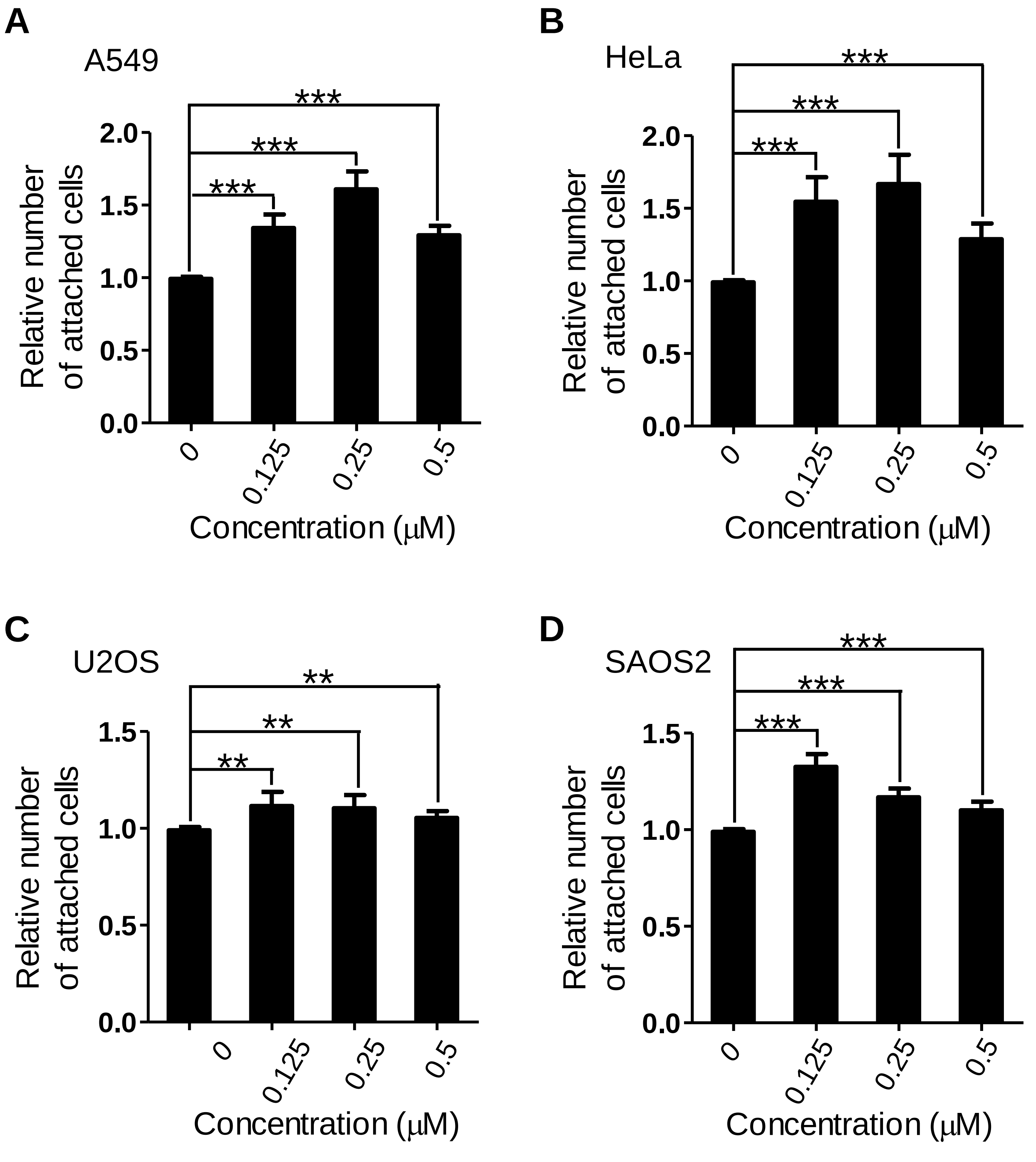


**Figure S1.** The effects of TPyP4-Pt on cell adhering to extracellular matrix. Cells were treated with TPyP4-Pt at indicated concentration and subjected to cell adhesion assay in which the cells attaching to extracellular matrix were determined. **A,** The effects of TPyP4-Pt on cell adhesion of A549. **B,** The effects of TPyP4-Pt on cell adhesion of HeLa. **C,** The effects of TPyP4-Pt on cell adhesion of U2OS. **D,** The effects of TPyP4-Pt on cell adhesion of SAOS2.

**A**

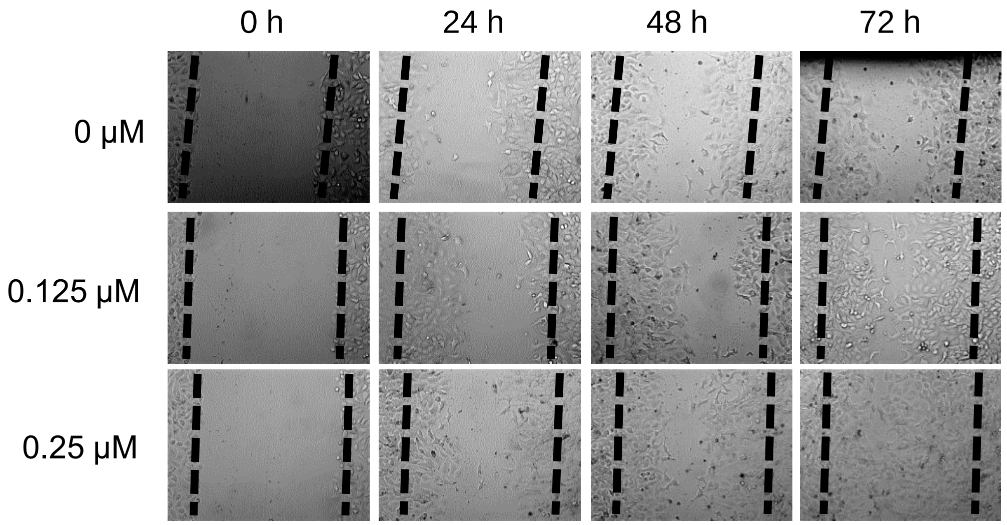


**B**

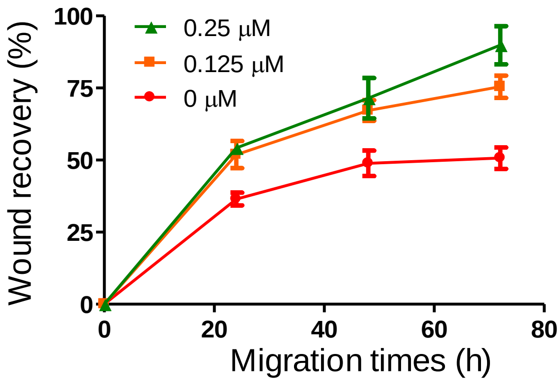


**Figure S2.** The effect of TPyP4-Pt on cell migration. The ability of cell migration was determined by scratch-wound healing assay. **A,** Scratch-wound healing assay on TPyP4-Pt-treated and untreated A549 cells. **B,** Quantification of **(A)**. Cell migration in the scratch area was calculated for TPyP4-Pt-treated A549 cells. Wound recovery was calculated for TPyP4-Pt-treated cells and untreated control. Values are average ± SD of three independent experiments.

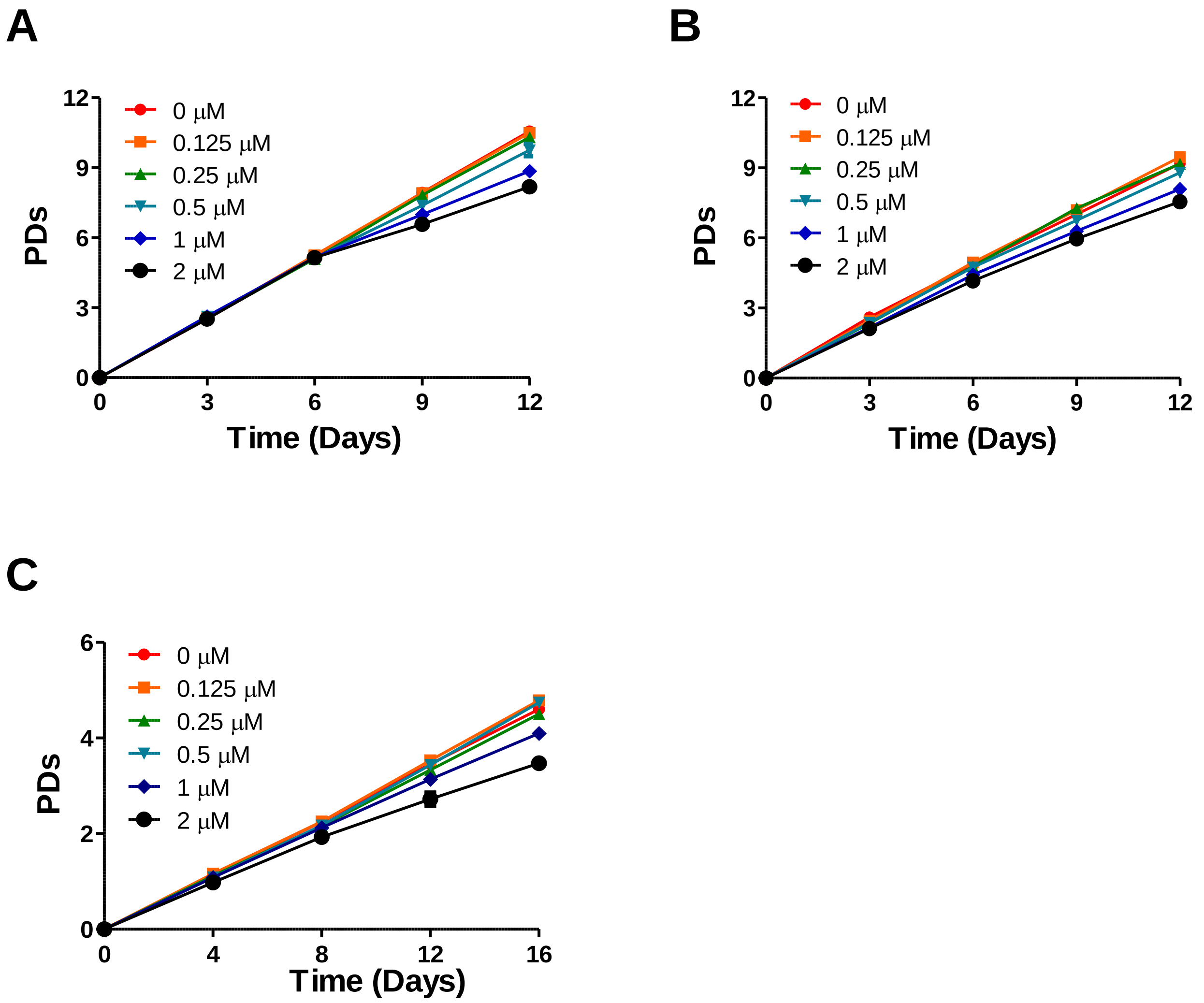


**Figure S3.** The effect of TMPyP4 on the proliferation of human cancer cells, including telomerase-positive HeLa cells **(A)** and telomerase-negative U2OS **(B)** and SAOS2 cells **(C)**.

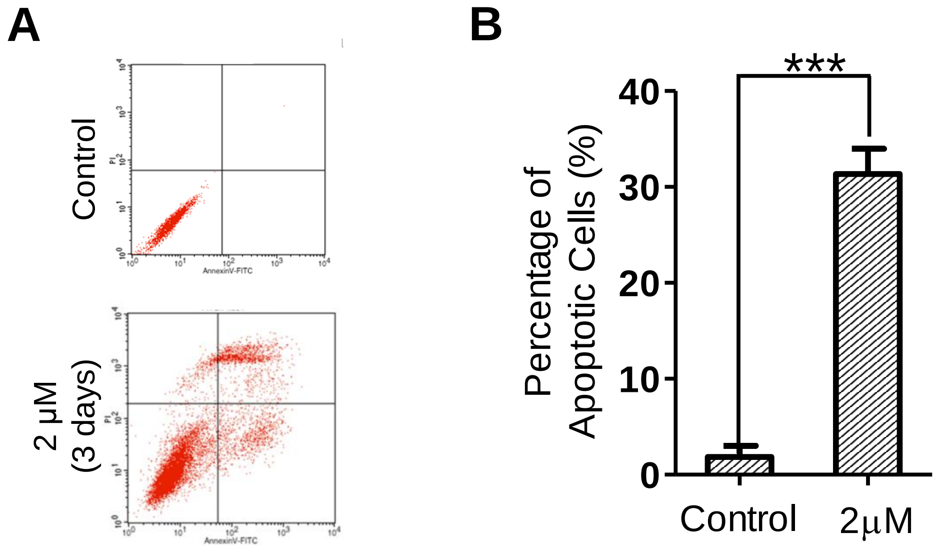


**Figure S4.** TMPyP4 treatment induces apoptosis of U2OS cancer cells. Apoptotic cells were assayed by Annexin V/PI staining and FACS analysis. **A,** U2OS cells were treated with TMPyP4 (2.0 µM). **B,** Quantification of (**A**). Values are average ± SD of three independent experiments.

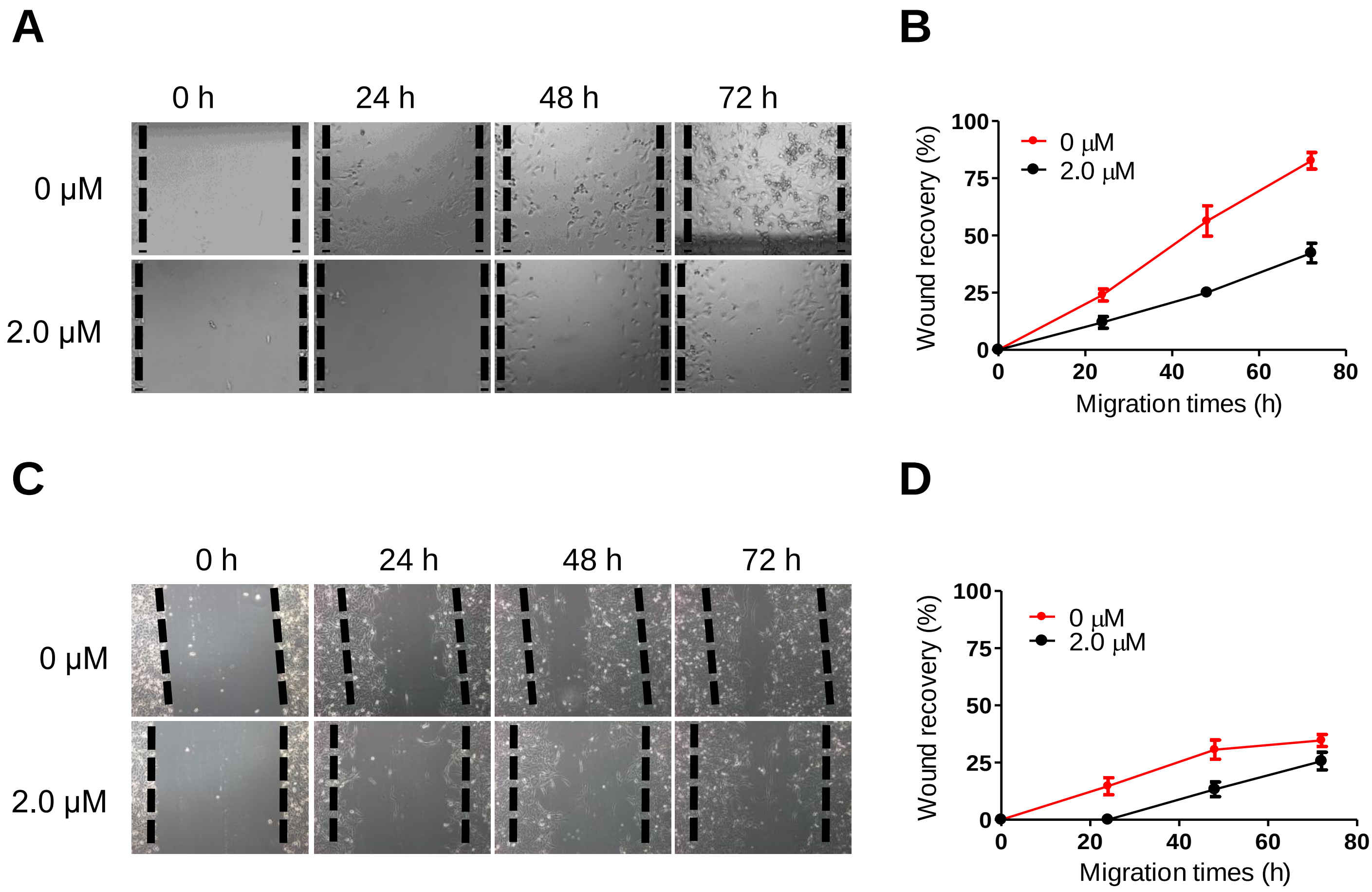


**Figure S5.** High-dose TMPyP4 suppresses cell migration. **A,** Scratch-wound healing assay on TMPyP4-treated and untreated HeLa cells. **B,** Quantification of **(A)**. **C,** Scratch-wound healing assay on TMPyP4-treated and untreated U2OS cells. **D,** Quantification of **(C)**. Cell migration in the scratch area was calculated for TMPyP4-treated cancer cells. Wound recovery was also calculated for TMPyP4-treated cells and untreated control. Values are average ± SD of three independent experiments.

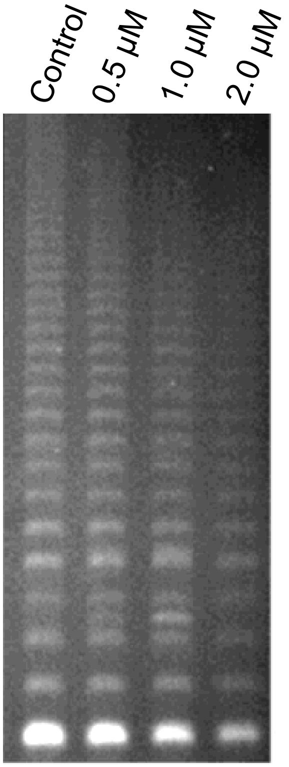


**Figure S6.** TRAP assay showed that TMPyP4 inhibits the telomerase activity in A549 cells.

**Table S1 Gene expression profile of TMPyP4-treated (+) (0.5 μM) and untreated A549 cells (list of top 100 changed genes).**

| GeneName | - | + | GeneName | - | + | GeneName | - | + | GeneName | - | + |
|---|---|---|---|---|---|---|---|---|---|---|---|
| NTS | 162 | 6046 | PCDH17 | 1 | 31 | KRT34 | 11 | 0 | B3GALT2 | 0 | 7 |
| MUC5B | 486 | 6274 | CCBE1 | 39 | 2 | CLEC19A | 11 | 0 | KLK2 | 0 | 7 |
| GREM1 | 350 | 3 | SPTSSB | 2 | 34 | NTSR1 | 11 | 0 | HIST2H2AB | 7 | 0 |
| CA2 | 17 | 241 | S100A9 | 0 | 25 | ADH4 | 0 | 9 | ALOX5AP | 7 | 0 |
| CHL1 | 6 | 178 | CDH16 | 0 | 22 | PCDH15 | 0 | 9 | LGI2 | 7 | 0 |
| UNC5D | 24 | 234 | PLCH2 | 20 | 0 | C4orf39 | 1 | 13 | SLC14A1 | 7 | 0 |
| BCHE | 12 | 169 | ITGA11 | 27 | 2 | A1CF | 1 | 13 | CAMK2B | 7 | 0 |
| TSPAN8 | 12 | 167 | NDNF | 0 | 16 | CXCR4 | 10 | 0 | C19orf69 | 0 | 6 |
| TF | 11 | 153 | EDN3 | 0 | 16 | LOC731223 | 1 | 12 | AZGP1 | 0 | 6 |
| SERPINA1 | 4 | 107 | COL14A1 | 2 | 23 | SERPINA10 | 1 | 12 | B3GNT6 | 0 | 6 |
| SPOCK1 | 117 | 7 | PGM5 | 16 | 0 | SLC6A14 | 0 | 8 | DSCAM-AS1 | 0 | 6 |
| CDH7 | 8 | 102 | LPHN3 | 0 | 14 | RAG1 | 0 | 8 | ANO4 | 0 | 6 |
| CADM2 | 1 | 66 | SLAMF7 | 15 | 0 | MTNR1A | 9 | 0 | CATSPERB | 0 | 6 |
| KCNH1 | 81 | 5 | LOC100505989 | 0 | 13 | SERTAD4 | 9 | 0 | RORC | 0 | 6 |
| PPARGC1A | 2 | 55 | CFI | 0 | 13 | LINC00235 | 8 | 0 | MIR637 | 6 | 0 |
| SEMA3D | 3 | 57 | TXNDC3 | 0 | 13 | LOC100133612 | 8 | 0 | DUSP26 | 6 | 0 |
| GPR141 | 0 | 42 | ALB | 0 | 13 | ARMCX1 | 8 | 0 | SNAI2 | 6 | 0 |
| HGF | 2 | 46 | CLDN7 | 0 | 13 | GJB4 | 8 | 0 | DIO3 | 6 | 0 |
| RASGEF1B | 2 | 44 | EDIL3 | 0 | 12 | ZDHHC22 | 8 | 0 | WFIKKN1 | 6 | 0 |
| SHISA3 | 4 | 49 | KRT23 | 0 | 11 | GNGT2 | 8 | 0 | SMTNL2 | 6 | 0 |
| LUM | 1 | 38 | PI15 | 0 | 11 | MAGEA6 | 8 | 0 | CILP | 6 | 0 |
| TM4SF5 | 4 | 48 | SYNDIG1 | 12 | 0 | IL1RL1 | 8 | 0 | ST8SIA1 | 6 | 0 |
| LY6K | 51 | 4 | TOX | 12 | 0 | VTCN1 | 8 | 0 | KCNE1 | 6 | 0 |
| FAM155A | 0 | 29 | CHST2 | 12 | 0 | C5orf47 | 0 | 7 | RGMA | 6 | 0 |
| F7 | 3 | 38 | LOC100128076 | 11 | 0 | RTBDN | 0 | 7 | SEMA3D | 3 | 57 |

**Table S2 Gene expression profile of TPyP4-Pt-treated (+) (0.5 μM) and untreated A549 cells (list of top 100 changed genes).**

| GeneName | - | + | GeneName | - | + | GeneName | - | + |
|---|---|---|---|---|---|---|---|---|
| LPHN3 | 0 | 35 | CFB | 47 | 763 | SERPINA3 | 31 | 309 |
| CDH16 | 0 | 32 | GRIK2 | 12 | 194 | CDH17 | 117 | 1151 |
| CFI | 0 | 51 | FGG | 181 | 2877 | RAB37 | 54 | 523 |
| SLC6A14 | 0 | 77 | FGB | 188 | 2818 | EGF | 3 | 29 |
| LOC100505989 | 0 | 48 | PRG4 | 5 | 73 | GJB1 | 3 | 29 |
| KLRC3 | 0 | 21 | SIDT1 | 20 | 279 | HABP2 | 6 | 56 |
| BPIFA1 | 0 | 28 | PPARGC1A | 2 | 27 | LOC100507567 | 4 | 37 |
| CLDN7 | 0 | 24 | DCDC5 | 16 | 213 | VIL1 | 6 | 54 |
| PCDH17 | 1 | 88 | FAAH2 | 2 | 26 | CFH | 434 | 3882 |
| KLRC2 | 1 | 85 | ADH6 | 8 | 103 | IFITM3 | 39 | 345 |
| PTPRB | 1 | 80 | C4BPA | 11 | 141 | ITIH2 | 13 | 115 |
| A1CF | 1 | 64 | CDH6 | 12 | 152 | PKIB | 15 | 132 |
| HGF | 2 | 111 | CHN2 | 3 | 38 | LCN2 | 40 | 350 |
| FER1L6 | 1 | 48 | PCDH10 | 5 | 63 | ROS1 | 7 | 61 |
| CPVL | 1 | 42 | HMCN1 | 8 | 98 | ANXA2P2 | 323 | 25 |
| TCN1 | 2 | 64 | FCGBP | 290 | 3515 | KLB | 40 | 3 |
| CAPN8 | 1 | 32 | ADH1C | 6 | 72 | GFRA1 | 274 | 19 |
| NR1H4 | 4 | 121 | UGT2B10 | 3 | 36 | CSNK2A1P | 44 | 3 |
| CADM2 | 1 | 28 | LBP | 3 | 36 | CCDC3 | 52 | 3 |
| RAB17 | 1 | 27 | TNFSF10 | 5 | 60 | ID4 | 707 | 39 |
| SERPINA1 | 4 | 105 | UGT2B11 | 9 | 106 | PLAC4 | 20 | 1 |
| CNR1 | 1 | 24 | NPY1R | 5 | 58 | ANKRD2 | 232 | 11 |
| LUM | 1 | 23 | KRT12 | 2 | 23 | PCDH11Y | 22 | 1 |
| IFITM1 | 2 | 45 | FGL1 | 179 | 2050 | STX11 | 24 | 1 |
| TF | 11 | 239 | RARRES1 | 119 | 1334 | CA8 | 574 | 24 |
| ATP8B5P | 3 | 65 | UGT2B15 | 6 | 67 | BCYRN1 | 3050 | 109 |
| SPTSSB | 2 | 42 | MUC16 | 3 | 33 | EDN2 | 34 | 1 |
| MUC5B | 484 | 10044 | FGA | 394 | 4293 | PSG4 | 43 | 1 |
| ELANE | 1 | 20 | F7 | 3 | 32 | MARCH4 | 698 | 13 |
| BPIFB1 | 4 | 77 | EPHA7 | 15 | 159 | CRX | 21 | 0 |
| TSPAN8 | 12 | 224 | FMO5 | 16 | 168 | SLC13A1 | 23 | 0 |
| CP | 266 | 4890 | LOC339535 | 3 | 31 | MIR663A | 27 | 0 |
| LRG1 | 2 | 34 | SETBP1 | 28 | 288 | GFRA1 | 274 | 19 |
| KRT20 | 7 | 115 | | | | | | |

**Table S3 Gene expression profile of TMPyP4-treated (+) (2.0 μM) and untreated A549 cells (list of top 100 changed genes).**

| GeneName | - | + | GeneName | - | + | GeneName | - | + |
|---|---|---|---|---|---|---|---|---|
| ABCC3 | 10209 | 18555 | MTRNR2L2 | 3689 | 2 | ID2 | 4181 | 1354 |
| AGR2 | 1213 | 3713 | MTRNR2L8 | 1909 | 3 | MRC2 | 2537 | 4374 |
| AGRN | 5760 | 10904 | NCL | 39424 | 15146 | SEMA4B | 1685 | 3318 |
| BCYRN1 | 3050 | 1 | NCOR2 | 4607 | 8875 | NOP58 | 4207 | 1390 |
| BHLHE40 | 3063 | 8929 | NR4A1 | 1987 | 9441 | IER3 | 2513 | 4311 |
| CNTN1 | 2679 | 7840 | PAPPA | 3384 | 7858 | SLTM | 4605 | 1650 |
| CXCL5 | 3532 | 6920 | PTGS2 | 2134 | 7551 | CNTNAP3B | 1212 | 2628 |
| DDIT4 | 4650 | 11574 | SIK1 | 4902 | 11841 | CELSR1 | 1922 | 3499 |
| DNAJA1 | 11347 | 2809 | SLC12A2 | 2675 | 5879 | LGALS3BP | 1230 | 2601 |
| DUSP1 | 3550 | 8583 | SLC23A2 | 1386 | 3579 | LRP10 | 2215 | 3811 |
| EGR1 | 2748 | 15621 | SMOX | 2588 | 5496 | COL4A5 | 1283 | 2637 |
| EIF5B | 8890 | 3332 | SQSTM1 | 24984 | 9948 | CHORDC1 | 3214 | 1052 |
| ELF3 | 4014 | 7400 | TGM2 | 2211 | 9221 | TCIRG1 | 1461 | 2679 |
| FN1 | 12856 | 44336 | THSD4 | 3009 | 7057 | CKS2 | 4039 | 1586 |
| FOS | 1821 | 6442 | TPM1 | 9306 | 3559 | PLA2G4A | 1227 | 2332 |
| GDF15 | 2023 | 4994 | SREBF1 | 3114 | 5672 | ESF1 | 2375 | 720 |
| GPX2 | 4135 | 7101 | GRAMD1A | 2581 | 4957 | CTNNAL1 | 2112 | 608 |
| HIF1A | 3155 | 7875 | RRM2 | 6012 | 2007 | RPL22L1 | 2439 | 792 |
| HMOX1 | 5896 | 1611 | HSPA1B | 2860 | 567 | EIF3J | 2943 | 1105 |
| HSP90AA1 | 101489 | 25240 | CD24 | 2476 | 4632 | PPIG | 2556 | 903 |
| HSPA1A | 4497 | 638 | HSPH1 | 6898 | 2619 | POLE3 | 2917 | 1103 |
| HSPA8 | 58262 | 12498 | NRP2 | 1518 | 3334 | UNC93B1 | 1252 | 2171 |
| HSPD1 | 35648 | 13124 | CEBPB | 1481 | 3277 | GPATCH4 | 2654 | 984 |
| HSPG2 | 2631 | 8129 | LRP5 | 2035 | 3974 | RRS1 | 2455 | 912 |
| IER2 | 2736 | 7260 | ERRFI1 | 2626 | 4657 | LOC220906 | 2002 | 683 |
| IGFBP4 | 26332 | 50831 | ALDH2 | 2078 | 3962 | PPIF | 2080 | 749 |
| ITGB4 | 2257 | 4826 | IGFBP3 | 2702 | 4701 | RSPO3 | 1954 | 686 |
| JUN | 1683 | 4263 | C11orf9 | 1278 | 2915 | DNTTIP2 | 2022 | 731 |
| JUP | 3750 | 7393 | PPYR1 | 1337 | 2988 | TIPARP | 1610 | 517 |
| KPNA2 | 16042 | 6297 | JUND | 1414 | 3068 | NSRP1 | 1593 | 558 |
| LAMA5 | 8652 | 20420 | TNRC18 | 2254 | 4120 | ADI1 | 1765 | 653 |
| LRP1 | 1321 | 3461 | SSB | 5638 | 2108 | DNAJC2 | 1602 | 567 |
| MAFK | 1359 | 3813 | LPGAT1 | 2923 | 4857 | APOC1 | 1205 | 364 |

**Table S4 "Go analysis" categorized genes changed in expression by TMPyP4 (0.5 μM) treatment in human cancer cells A549.**

| Term | PValue | Genes |
|---|---|---|
| **GO:0042060~wound healing** | 0.0000 | SERPINA10, ERBB3, C4BPB, F7, COL5A1, FGG, FGA, F5, FGB, SERPINA1, IGFBP1, ENTPD2, TM4SF4 |
| **GO:0009611~response to wounding** | 0.0001 | TF, ACHE, SERPINA10, ERBB3, S100A9, CHST2, C4BPB, F7, COL5A1, FGG, F5, FGA, CCL20, CXCR4, FGB, CFH, SERPINA1, RTN4RL2, CFI, IGFBP1, ENTPD2, TM4SF4 |
| **GO:0007155~cell adhesion** | 0.0005 | CLDN7, ACHE, SVEP1, PCDH11X, ITGA11, SPOCK1, PCDH15, SLAMF7, EDIL3, PCDH17, CDH4, COL5A1, AZGP1, CDH7, PGM5, COL14A1, CDH16, F5, CLDN2, LAMC2, FCGBP, CHL1, MUC5B, MUC16 |
| **GO:0022610~biological adhesion** | 0.0005 | CLDN7, ACHE, SVEP1, PCDH11X, ITGA11, SPOCK1, PCDH15, SLAMF7, EDIL3, PCDH17, CDH4, COL5A1, AZGP1, CDH7, PGM5, COL14A1, CDH16, F5, CLDN2, LAMC2, FCGBP, CHL1, MUC5B, MUC16 |

**Table S5 "Go analysis" categorized genes changed in expression by TPyP4-Pt (0.5 μM) treatment in human cancer cells A549.**

| Term | PValue | Genes |
|---|---|---|
| **GO:0009611~response to wounding** | 2.28E-12 | CXCL1, TF, ACHE, C3, ERBB3, TNC, MST1, BDKRB1, C1S, BDKRB2, CCL5, TPM1, CFHR1, FGG, HMCN1, FGA, CCL20, FGB, PAX7, SERPINE1, SERPINA3, CFH, APOH, SERPINA1, CFI, LBP, SCNN1B, ENTPD2, FN1, CFB, EFEMP2, F7, C4BPA, UNC13D, HIF1A, ADM, PLSCR4, F2, TFPI, TM4SF4, CD14 |
| **GO:0042060~wound healing** | 4.15E-09 | ERBB3, EFEMP2, MST1, F7, TPM1, FGG, HMCN1, HIF1A, PLSCR4, FGA, FGB, PAX7, F2, SERPINE1, APOH, TFPI, SERPINA1, SCNN1B, ENTPD2, TM4SF4, FN1 |
| **GO:0007155~cell adhesion** | 0.004027503 | CLDN7, PLXNC1, ACHE, TNC, COL28A1, ITGA10, CCL5, CDH4, CDH6, LGALS3BP, CEACAM1, FN1, SVEP1, PCDH11Y, PCDH10, ITGA1, PCDH17, MCAM, GPR98, CDH16, CDH17, CLDN2, CNTN1, FCGBP, MUC5B, HABP2, MUC16 |
| **GO:0022610~biological adhesion** | 0.004032231 | CLDN7, PLXNC1, ACHE, TNC, COL28A1, ITGA10, CCL5, CDH4, CDH6, LGALS3BP, CEACAM1, FN1, SVEP1, PCDH11Y, PCDH10, ITGA1, PCDH17, MCAM, GPR98, CDH16, CDH17, CLDN2, CNTN1, FCGBP, MUC5B, HABP2, MUC16 |

**Table S6 "Go analysis" categorized genes changed in expression by TMPyP4 (2.0 μM) treatment in human cancer cells A549.**

| Term | PValue | Genes |
|---|---|---|
| **GO:0042060~wound healing** | 8.59E-05 | NOTCH3, FGG, HIF1A, HNF4A, FGA, ERBB3, FGB, GSN, HMOX1, ANXA8L2, IGFBP1, TPM1, ENTPD2, TM4SF4, FN1 |
| **GO:0030155~regulation of cell adhesion** | 2.23E-04 | RND1, IL8, ERBB3, GSN, LAMA5, SMOC1, TGM2, CELSR2, COL1A1, CD24, SERPINI1, TPM1 |
| **GO:0016477~cell migration** | 4.16E-04 | NRP2, IL8, CXCL3, NR4A2, DCDC2, CDH2, CCL5, HIF1A, CTGF, LAMA5, BTG1, SEMA3F, PRSS3, CD24, NR2F1, FN1, LRP5 |
| **GO:0048870~cell motility** | 4.70E-04 | NRP2, IL8, CXCL3, NR4A2, DCDC2, CDH2, CCL5, HIF1A, CTGF, LAMA5, BTG1, SEMA3F, PRSS3, DNAJA1, CD24, NR2F1, FN1, LRP5 |
| **GO:0042127~regulation of cell proliferation** | 1.04E-05 | CXCL1, RARRES1, CXCL5, PTGS2, ERBB3, TNFSF15, BDKRB2, SOX9, HMOX1, TGM2, KRT4, CD24, FGFBP1, BMP4, TCIRG1, KLF5, TP53I11, IRS2, IL8, CRIP2, HES1, CD38, PLA2G4A, HIF1A, ATF3, NUPR1, HNF4A, ID2, BTG1, LAMA5, JUN, MNT, ID4, TBX18, IGFBP3, ADRA1D, TM4SF4, LRP5 |
| **GO:0010941~regulation of cell death** | 5.15E-05 | IER3, PTGS2, ERBB3, CBX4, TNFSF15, CD70, CDH1, HSPA1A, BDKRB2, HSPA1B, SOX9, SQSTM1, HMOX1, TGM2, BCL3, CD24, ANGPTL4, BMP4, CEBPB |
| **GO:0042981~regulation of apoptosis** | 8.84E-05 | IER3, PTGS2, ERBB3, CBX4, TNFSF15, CD70, CDH1, HSPA1A, BDKRB2, HSPA1B, SOX9, SQSTM1, HMOX1, TGM2, BCL3, CD24, ANGPTL4, CEBPB, SOCS3, BCL2A1, NR4A2, NR4A1, PDE3A, FURIN, PPIF, CD38, PLA2G4A, NUPR1, DUSP1, BTG1, BBC3, JUN, MAP3K10, ERN1, MNT, HSPD1, IGFBP3 |
| **GO:0043067~regulation of programmed cell death** | 1.07E-04 | IER3, PTGS2, ERBB3, CBX4, TNFSF15, CD70, CDH1, HSPA1A, BDKRB2, HSPA1B, SOX9, SQSTM1, HMOX1, TGM2, BCL3, CD24, ANGPTL4, CEBPB, SOCS3, BCL2A1, NR4A2, NR4A1, PDE3A, FURIN, PPIF, CD38, PLA2G4A, NUPR1, DUSP1, BTG1, BBC3, JUN, MAP3K10, ERN1, MNT, HSPD1, IGFBP3 |
